# Impact of point spread function modelling and time of flight on FDG uptake measurements in lung lesions using alternative filtering strategies

**DOI:** 10.1186/s40658-014-0099-3

**Published:** 2014-11-30

**Authors:** Ian S Armstrong, Matthew D Kelly, Heather A Williams, Julian C Matthews

**Affiliations:** Nuclear Medicine, Central Manchester University Hospitals, Oxford Road, Manchester, UK; Institute of Population Health, MAHSC, University of Manchester, Manchester, UK; Molecular Imaging, Healthcare Sector, Siemens PLC, Oxford, UK

**Keywords:** PET quantification, PSF modelling, Time-of-flight, SUV, Total lesion glycolysis

## Abstract

**Background:**

The use of maximum standardised uptake value (SUV_max_) is commonplace in oncology positron emission tomography (PET). Point spread function (PSF) modelling and time-of-flight (TOF) reconstructions have a significant impact on SUV_max_, presenting a challenge for centres with defined protocols for lesion classification based on SUV_max_ thresholds. This has perhaps led to the slow adoption of these reconstructions. This work evaluated the impact of PSF and/or TOF reconstructions on SUV_max_, SUV_peak_ and total lesion glycolysis (TLG) under two different schemes of post-filtering.

**Methods:**

Post-filters to match voxel variance or SUV_max_ were determined using a NEMA NU-2 phantom. Images from 68 consecutive lung cancer patients were reconstructed with the standard iterative algorithm along with TOF; PSF modelling - Siemens HD·PET (HD); and combined PSF modelling and TOF - Siemens ultraHD·PET (UHD) with the two post-filter sets. SUV_max_, SUV_peak_, TLG and signal-to-noise ratio of tumour relative to liver (SNR_(T-L)_) were measured in 74 lesions for each reconstruction. Relative differences in uptake measures were calculated, and the clinical impact of any changes was assessed using published guidelines and local practice.

**Results:**

When matching voxel variance, SUV_max_ increased substantially (mean increase +32% and +49% for HD and UHD, respectively), potentially impacting outcome in the majority of patients. Increases in SUV_peak_ were less notable (mean increase +17% and +23% for HD and UHD, respectively). Increases with TOF alone were far less for both measures. Mean changes to TLG were <10% for all algorithms for either set of post-filters. SNR_(T-L)_ were greater than ordered subset expectation maximisation (OSEM) in all reconstructions using both post-filtering sets.

**Conclusions:**

Matching image voxel variance with PSF and/or TOF reconstructions, particularly with PSF modelling and in small lesions, resulted in considerable increases in SUV_max_, inhibiting the use of defined protocols for lesion classification based on SUV_max_. However, reduced partial volume effects may increase lesion detectability. Matching SUV_max_ in phantoms translated well to patient studies for PSF reconstruction but less well with TOF, where a small positive bias was observed in patient images. Matching SUV_max_ significantly reduced voxel variance and potential variability of uptake measures. Finally, TLG may be less sensitive to reconstruction methods compared with either SUV_max_ or SUV_peak_.

**Electronic supplementary material:**

The online version of this article (doi:10.1186/s40658-014-0099-3) contains supplementary material, which is available to authorized users.

## Background

[^18^F]2-Fluoro-2-deoxy-d-glucose (FDG) positron emission tomography (PET) has been shown to play a key role in the management of patients with non-small cell lung cancer in terms of staging and prognosis [1]-[5] and monitoring response to therapy [6]. In these applications, the uptake of FDG expressed as standardised uptake value (SUV) is of key importance, with SUV_max_ being the most commonly reported measure [7]. The use of SUV_max_ for discrimination between benign and malignancy for soft tissue masses and lymph nodes has been demonstrated for lung cancer patients [8],[9] and changes in SUV_max_ used as an indicator of response to therapy [10].

While the use of SUV_max_ is commonplace, it is known to be sensitive to both reconstruction parameters [11] and the amount of statistical image noise, leading to poorer test-retest consistency relative to other SUV-based metrics [12],[13]. Consequently, alternative metrics such as SUV_peak_ [14] and total lesion glycolysis (TLG), the product of SUV_mean_ and metabolic tumour volume derived from the PET images, have been suggested for use, particularly in monitoring response to therapy [6],[15]. Recently, TLG has also been shown to offer superior prognostic information than SUV_max_ [16]-[20].

In recent years, there have been significant advances in iterative image reconstruction algorithms and scanner hardware. Consequently, reconstruction algorithms that include point spread function (PSF) modelling [20],[21] and time of flight (TOF) [22] have become commercially available on PET/CT scanners, with TOF also available on PET/MR [23].

The use of PSF modelling, with and without TOF, has been shown to improve signal-to-noise ratio (SNR) [24]-[27] and lesion detectability [28]-[30] partly through decreasing voxel variance. However, the implementation of PSF modelling, both within projection space and image space, from different manufacturers and also academic institutions has been shown to produce Gibbs artefacts [21],[31]-[35] (Nick Vennart, personal communication). In patient imaging, the Gibbs artefact, combined with reduced partial volume effects, has a significant impact on SUV_max_ [36]-[38]. This is particularly evident with minimal or no post-reconstruction filtering, which has been shown in phantom studies with numerical observers to provide greater lesion detectability [28]-[30]. Changes to SUV_max_ as a consequence of PSF modelling present a challenge as changes to defined local practice for reporting may be required such as changing the thresholds used for the discrimination of malignancy. The scanner used in this study has been part of a multi-site network of scanners for routine FDG oncology imaging since 2009. SUV_max_ is the reported uptake metric, and the consensus amongst local reporting clinicians within the network is that lesions with SUV_max_ > 5.0 are considered highly suspicious of malignant disease.

It is necessary, in practice, to smooth clinical images to provide image quality that is deemed acceptable for clinical reporting. This degrades the spatial resolution but increases signal to noise. The degree of smoothing applied at any given centre is heavily influenced by the experience and personal preferences of the reporting clinicians, informed by the advice of physicists providing scientific support. Where several PET scanners serve the same patient population, it is also advantageous to match imaging performance across the network in terms of visual image quality and quantitative characteristics.

A trade-off curve of signal enhancement versus noise reduction when using PSF and/or TOF algorithms can be established by applying a range of reconstruction post-filters. It has been demonstrated that it is possible to match SUV_max_ from PSF-based reconstruction with traditional non-PSF algorithms by applying a particular post-filter. Lasnon et al. [39] showed that a 7.0-mm full-width-half-maximum (FWHM) post-filter with PSF reconstruction gave comparable recovery coefficients in phantom data to non-PSF reconstructions and brought the recovery coefficients in line with European recommendations [40]. Another study proposed the application of a post-filter for the purpose of quantification [41]. This study also demonstrated that despite a spatially dependent PSF, this approach of using a single post-filter choice was adequate for all lesions irrespective of their location in the field of view. The application of a relatively broad post-filter to PSF modelling images may seem counterintuitive as it will undo the improvements in partial volume effect, but there are likely to be other benefits that have not been reported such as a reduction in voxel variance in the images.

Another potential solution may be to use alternative uptake metrics to SUV_max_. One study [37] suggested that TLG may be more stable when comparing PSF to non-PSF reconstruction, but this study only assessed ten lung lesions. Another study [38] has suggested the move to SUV_mean_ based upon a 50% isocontour of SUV_max_. To our knowledge, there are currently no studies that investigate the impact of these reconstructions with PSF modelling and TOF on TLG and SUV_peak_.

The primary aim of this study was to evaluate the impact of PSF modelling and TOF on SUV_max_-based lesion classification as implemented at the local institution. This was performed using Siemens reconstruction software including implementations for TOF and PSF modelling (HD, UHD). Implementations of reconstruction algorithms can differ, and therefore, the results might be specific to HD and UHD; however, we feel it is likely that findings may be generalisable to other reconstruction implementations with similar philosophies. Any change in FDG uptake measurements across different reconstruction protocols can hopefully allow other centres to assess how such changes may impact their approaches to lesion classification. Two set criteria for post-filtering the images were assessed based upon characteristic locations on a signal enhancement versus noise reduction trade-off curve. These two points are 1) matching image noise (voxel variance) which was expected to enhance signal and 2) matching signal (SUV_max_) which, based on previous studies [39],[41], was anticipated to require greater levels of post-filtering and hence reduce image noise. This latter approach is aimed to be particularly relevant to centres that wish to maintain uptake quantification for practical purposes, which is particularly important in multi-site imaging networks. In addition, this work aimed to expand on the results of previous studies [36]-[38] with the addition of TOF, evaluation of other uptake metrics such as SUV_peak_ and TLG, and determining gains in SNR for the two strategies.

## Methods

### PET/CT scanner

The PET scanner used in this study was a Siemens Biograph mCT with 64 slice CT (Siemens Medical Solutions, Erlangen, Germany). The scanner has a four-ring extended axial field of view of 21.6 cm (TrueV) and includes options for PSF modelling (Siemens HD·PET) and combined PSF modelling with TOF (Siemens ultraHD·PET) in the image reconstruction. Performance data for the scanner has been published previously [42].

### Phantom acquisitions

A NEMA NU-2 image quality (IQ) phantom (PTW, Freiburg, Germany) was filled with [^18^F]FDG so that the background compartment and all six hot spheres had activity concentrations of 5.19 and 41.7 kBq/ml, respectively. This 8:1 contrast was chosen to mimic lung lesion contrast, which is generally high. In order to divide the data into ten replicate datasets, a gated 60-min list-mode acquisition was performed using an ECG simulator as the gating input. Each replicate image contained 30 million (±0.2%) net true coincidences as this was typical of the number of counts measured over the thorax in our standard patient acquisitions. Images were reconstructed using four methods: standard 3-D ordinary Poisson ordered subset expectation maximisation (OSEM) reconstruction; OSEM with TOF (TOF); OSEM with PSF modelling - Siemens HD·PET (HD); and OSEM with both PSF and TOF - Siemens ultraHD·PET (UHD). For non-TOF reconstructions, 3 iterations and 24 subsets (3i24s) were used, while for TOF reconstructions, 2 iterations and 21 subsets (2i21s) were used.

Two iterations were chosen for TOF reconstructions as TOF has been shown to provide faster convergence with comparable signal to noise achieved in fewer iterations than non-TOF [27],[43], and it has been shown in published performance data for the scanner that one fewer iteration with TOF is optimal [42], providing similar background variability and marginally superior contrast recovery in smaller objects. However, it is not possible to exactly match the number of subsets for TOF and non-TOF reconstructions. All images were reconstructed into a 256 × 256 matrix with voxel sizes of 3.2 mm × 3.2 mm × 2.0 mm. As is routinely performed with patient data, a 5.0-mm FWHM Gaussian post-filter was applied to the OSEM images. The baseline parameters of 3 iterations and 24 subsets and 5.0-mm post-filter for OSEM reconstruction have been in routine use since the scanner was commissioned in 2009. These parameters were selected to align SUV_max_ quantification and voxel variance with other scanners in the local oncology imaging network.

A variety of post-filters with different kernel widths was applied to the TOF, HD and UHD images with kernel widths ranging from 0 to 10 mm FWHM in step sizes for 0.1 mm.

#### Noise matching

Twelve circular regions of interest (ROIs) of 37-mm diameter were placed in the phantom background over five separate slices (60 ROIs in total) of the IQ phantom image in accordance with the NEMA NU-2-2007 standard [44]. For each image replicate, the average coefficient of variation (COV) over the 60 ROIs was calculated as

1 COVR=∑k=160σk,Rμk,R,

where *σ*_*k,R*_ and *μ*_*k,R*_ are the voxel standard deviation and mean, respectively, within ROI *k* and replicate *R*. The mean and standard deviation of COV_*R*_ was determined across all ten replicate images. The OSEM 3i24s 5.0-mm post-filter image was used to compute the reference COV value. For the three other reconstruction methods, the post-filter that gave the smallest difference in COV, relative to the OSEM image, was determined.

#### SUV_max_ matching

SUV_max_ is the uptake measure used in our routine patient reports and so was the measure chosen to match across the reconstruction algorithms. To achieve this, SUV_max_ was measured in each hot sphere in the phantom for the OSEM images using a 3-D volume of interest, equal in diameter to each true sphere size and centred on the sphere. As with the COV matching, a post-filter was incremented in 0.1-mm steps on the other three reconstructions until the summed squared difference of SUV_max_ for the six hot spheres relative to those in the OSEM image was minimised.

### FDG patient acquisitions

#### Patient preparation

Retrospective data from 68 (33 males; mean [range] weight: 72.5 kg [40 to 136]; mean [range] body mass index: 26.3 kg/m^2^ [14.1 to 51.8]) consecutive routine oncology patients referred for assessment of single pulmonary nodule or staging of non-small cell lung cancer were included in this study. All data were fully anonymised before inclusion. Patients fasted for 6 h prior to the injection of FDG and were asked to drink at least 500 ml of water before the scan. Blood glucose was measured with permissible limits of 3.0 to 12.5 mmol/l. Patients with a body weight <100 kg were prescribed 350 MBq of [^18^F]FDG, while those with body weight >100 kg (two in this study) were prescribed 400 MBq. The mean [range] administered activity of [^18^F]FDG was 365.5 MBq [242.0 to 423.1]. It can be noted that the minimum dose administered is considerably below the prescribed activity - this was due to a patient arriving late and insufficient remaining activity in the stock vial. The mean [range] time was 64.3 [59 to 87] min from the time of injection to commencing the scan. Advice from the local ethics committee deemed that the use of retrospective anonymised patient data did not require formal ethical approval.

#### PET/CT acquisitions

The PET acquisition was performed from eyes to mid-thigh for all patients, requiring six or seven bed positions. The acquisition time for each bed position was 2.5 min. Attenuation correction was performed using a non-contrast CT acquisition performed prior to the PET acquisition. Scatter and random corrections were applied to all images. All images were reconstructed with OSEM 3i24s and 5.0-mm post-filter as the reference, along with the phantom-determined TOF, HD and UHD protocols, which match either voxel COV or SUV_max_.

#### Uptake measurements

All images were viewed and the uptake quantified using Siemens TrueD image display software (Siemens Medical Solutions, Erlangen, Germany). In each patient, a 3-cm-diameter spherical volume of interest (VOI) was placed within an area of uniform FDG distribution in the liver, and the COV of the voxels within the VOI was calculated. Three FDG uptake measurements were derived for each identified lesion within the lung: SUV_max_, SUV_peak_ (as defined in the PET response criteria in solid tumours (PERCIST) protocol [14]) and TLG. SUV was normalised to patient body weight only. Volume delineation for TLG was performed using a 40% threshold of SUV_max_ (TLG-40). Recent meta-analyses [16],[17] have highlighted several methods for volume delineation - either using percentage or absolute SUV thresholds. The choice of a percentage threshold in this study was based on a hypothesis that as the magnitude of the partial volume effect varied with different reconstructions, the impact on the tumour volume and SUV_mean_ would be inversely related. This may result in a more stable value for the TLG. It should be noted that other methods of delineation are likely to produce alternative results. Lesion volume was measured on the OSEM image using a 40% threshold of SUV_max_.

#### Signal to noise

It is difficult to estimate SNR directly in a lesion due to inhomogeneous uptake; therefore, we have adopted the use of the liver as a source for the background and noise measurement. This technique has been performed previously [25] and is considered a reasonable relative surrogate for SNR in the lesion. For lesions with SUV_max_ above the PERCIST threshold of 1.5 times the mean SUV in the liver VOI + 2 standard deviations of the voxels within the liver VOI [14], the signal-to-noise ratio of the tumour, relative to the liver, (SNR_(T-L)_) was calculated as

2 $${SNR}_{\left(T‐L\right)}=\frac{\mathrm{Tumour}-\mathrm{Liver}}{\sigma_{L}}\text{,}$$

where the *Tumour* refers to SUV_max_ in the lung lesion, *Liver* is the mean SUV measured in the liver VOI and *σ*_L_ is the standard deviation of voxel values measured in the liver VOI. This method allows comparison to other studies, which have used the same metric [25],[42]. SNR_(T-L)_ of all qualifying lesions was determined for each reconstruction using the two filtering schemes of matched voxel COV and matched SUV_max_. The gain in SNR_(T-L)_ was expressed for the TOF, HD and UHD reconstructions as the ratio to the SNR_(T-L)_ measurements from the standard OSEM images of the same patient.

#### Statistical analysis

Relative percentage differences of the uptake metrics relative to OSEM were expressed as mean with 95% confidence intervals. Bland-Altman analysis was also performed on the data. Relative changes of >25% for SUV_max_ and >30% for SUV_peak_ were considered clinically significant based upon EORTC [10] and PERCIST [14] guidelines respectively. In addition, hypothetical changes to patient management as a consequence of SUV_max_ based on local practice were recorded. Differences in voxel COV in the liver VOI and gains in SNR_(T-L)_ were assessed using a paired *t* test with a *p* value <0.05 considered to be significant.

## Results

### Phantom images

**Figure 1:**
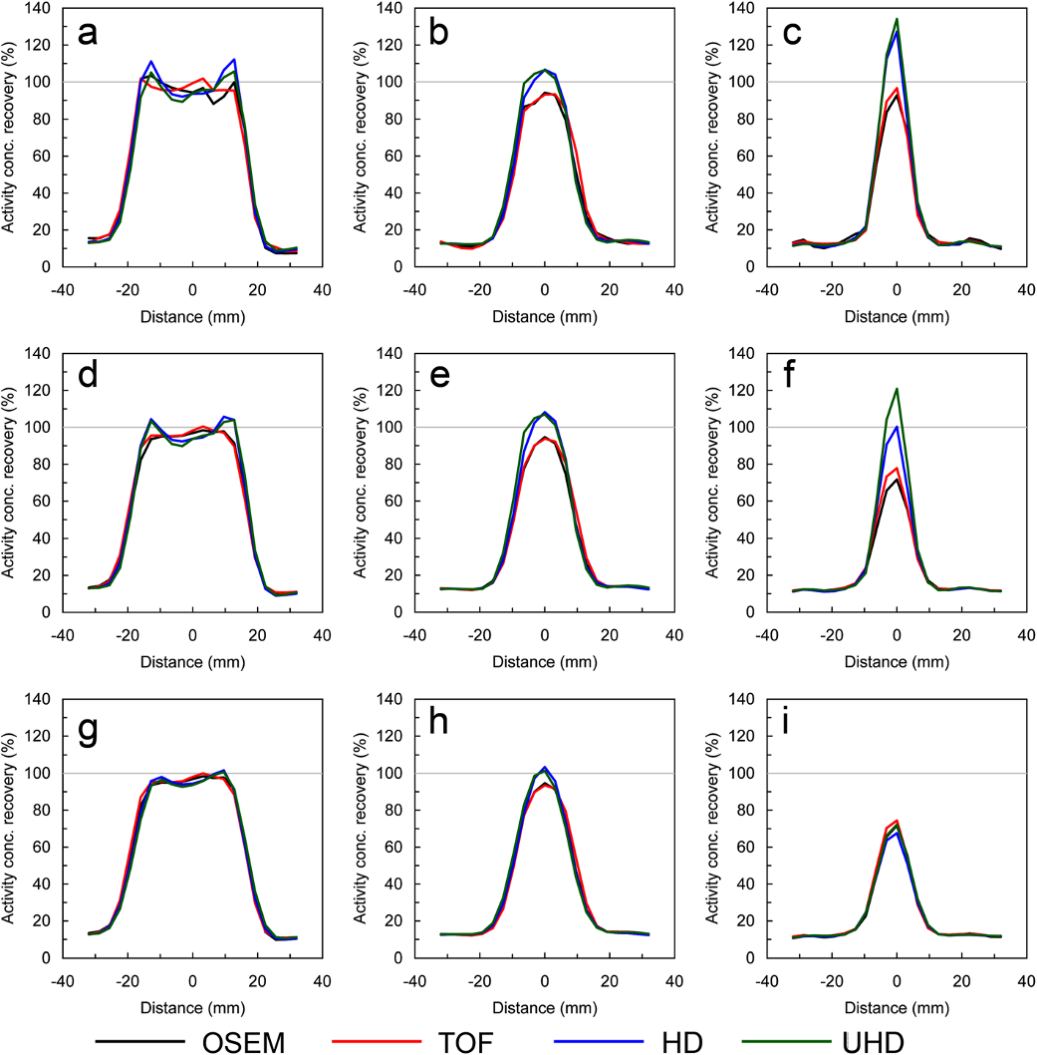
**Phantom sphere profiles.** Transaxial line profiles through the centre of the 37-mm sphere **(a, d, g)**, 22-mm sphere **(b, e, h)** and 13-mm sphere **(c, f, i)**. Plots in the top row are for unfiltered images, those in the centre row are for post-filters to match voxel COV, and those in the bottom row are for plots to match SUV_max_.

The FWHM of the post-filters obtained for matching voxel COV to OSEM 3i24s and a 5.0-mm post-filter were 4.4, 3.8 and 2.9 mm for TOF, HD and UHD, respectively. The FWHM of the post-filters obtained for matching SUV_max_ were 4.8, 6.6 and 6.5 mm for TOF, HD and UHD, respectively. To provide an illustration of the underlying impact of each algorithm, SUV_max_, expressed as a percentage of the true activity concentration, and noise data are first shown with no post-filter in Table 1. Data are then presented with the two post-filter sets as described in Table 2. From the data, it is seen that there is considerable increase in SUV_max_ in the two smallest spheres with HD and UHD with matched voxel COV. The variability of SUV_max_ was greater in the two smallest spheres at matched voxel COV, particularly with HD and UHD; the positive bias in the larger spheres with OSEM and TOF at matched voxel COV is likely to be due to image voxel variance, while with HD and UHD at matched voxel COV, Gibbs artefacts are also expected to contribute. This can be seen in Figure 1, which shows profiles through the centre of the 37-, 22- and 13-mm spheres.

**Table 1:** **Phantom recovery data for unfiltered images**

	Sphere size						Background voxel COV
	10 mm	13 mm	17 mm	22 mm	28 mm	37 mm	
OSEM (%)	106 (19.5)	136 (20.2)	155 (19.8)	168 (18.0)	184 (9.5)	186 (8.2)	46.3 (0.51)
TOF (%)	90 (12.0)	123 (11.2)	132 (7.2)	144 (11.0)	157 (11.4)	167 (13.7)	37.4 (0.48)
HD (%)	99 (13.7)	155 (7.2)	144 (6.2)	145 (5.3)	144 (7.1)	147 (6.0)	18.2 (0.32)
UHD (%)	103 (9.0)	151 (8.8)	141 (5.9)	136 (4.5)	135 (5.9)	138 (5.6)	14.9 (0.46)

SUV_max_ in each of the image quality spheres expressed as a percentage of the true activity concentration, and voxel COV in the phantom background. Data are shown for all four reconstruction algorithms with no post-filtering applied. Values are mean and standard deviation (SD) obtained from the replicates, with the latter shown in parentheses. For clarity, the SD shown is the SD across the replicates expressed as a percentage of the true activity concentration in the sphere.

**Table 2:** **Phantom recovery data**

	Sphere size						Background voxel COV
	10 mm	13 mm	17 mm	22 mm	28 mm	37 mm	
OSEM	53	80	96	102	110	110	12.9
Matched voxel COV							
TOF (%)	58 (4.4)	84 (3.4)	97 (3.2)	101 (2.4)	110 (2.4)	113 (3.8)	12.8 (0.23)
HD (%)	73 (7.1)	123 (4.5)	122 (2.9)	121 (5.3)	121 (3.8)	123 (2.8)	12.8 (0.38)
UHD (%)	89 (6.6)	138 (7.5)	129 (3.1)	123 (2.7)	125 (4.0)	127 (4.2)	12.7 (0.43)
Matched SUV_max_							
TOF (%)	55 (3.8)	80 (2.8)	94 (2.9)	98 (1.8)	107 (2.3)	110 (2.9)	11.2 (0.37)
HD (%)	47 (3.0)	80 (2.3)	103 (2.7)	102 (3.5)	105 (1.3)	106 (0.9)	7.57 (0.17)
UHD (%)	49 (1.9)	81 (1.8)	102 (2.0)	100 (1.2)	104 (2.0)	105 (1.2)	6.27 (0.33)

SUV_max_ in each of the image quality spheres expressed as a percentage of the true activity concentration, and voxel COV in the phantom background. Data are shown for OSEM (reference reconstruction) and the PSF and TOF-based reconstructions with the two post-filter sets. Values are mean and standard deviation (SD) obtained from the replicates, with the latter shown in parentheses. For clarity, the SD shown is the SD across the replicates expressed as a percentage of the true activity concentration in the sphere.

With post-filters to match SUV_max_ recovery, variability is comparable or less with HD and UHD compared with OSEM. To verify the cross-calibration between the dose calibrator and scanner, the activity concentration, averaged across the 60 background ROIs, was measured as 5.14 ± 0.1 kBq/ml.

### Patient images

**Figure 2:**
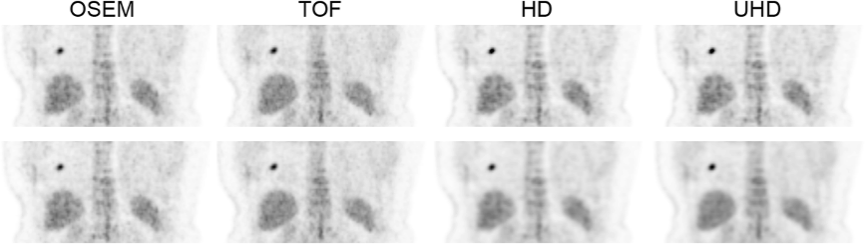
**Coronal PET images.** Coronal images from a female patient with BMI 37 kg/m^2^. Top row: images with matched voxel COV. Bottom row: images with matched SUV_max_.

Figure 2 shows images from a single representative female patient with a BMI of 37 kg/m^2^. The image has been cropped to show only the lung lesion and liver. Voxel COV within the liver VOI was 16.3%, 15.0%, 16.5% and 15.4% for OSEM, TOF, HD and UHD, respectively, with matched voxel COV post-filters and 13.5%, 10.8% and 7.95% for TOF, HD and UHD, respectively, with matched SUV_max_ post-filters. SUV_max_ for the lesion in the right lung was 5.4, 6.0, 8.2 and 10.1 for OSEM, TOF, HD and UHD, respectively, with matched noise post-filters and 5.2, 5.7 and 5.7 for TOF, HD and UHD, respectively, with matched SUV_max_ post-filters. The visual reduction in voxel variance within the liver is evident in the HD and UHD images with the matched SUV_max_ protocol.

#### Liver noise

Table 3 shows the voxel COV data measured in the VOI within the patient livers. There were no significant differences for the PSF and TOF-based reconstructions versus OSEM when using the matched voxel COV post-filters. As with the phantom data, significant reductions of voxel COV were measured for PSF and TOF-based reconstructions compared with OSEM using the post-filters to match SUV_max_ recovery. The mean measurements of voxel COV in the liver VOI for TOF, HD and UHD were 90%, 65% and 56%, respectively, of the value measured using OSEM.

**Table 3:** **Patient liver noise**

Liver COV (%)	OSEM	TOF	HD	UHD
Matched COV	10.6 (2.3)	10.5 (2.4)	10.6 (2.2)	10.4 (2.1)
Matched SUV_max_		9.5 (2.1)	6.8 (1.5)	5.9 (1.3)

Image noise, expressed as coefficient of variation (COV), measured in the liver for each reconstruction for matched voxel COV and matched SUV_max_ post-filters. Values are mean and standard deviation, with the latter shown in parentheses.

#### FDG uptake measurements

**Figure 3:**
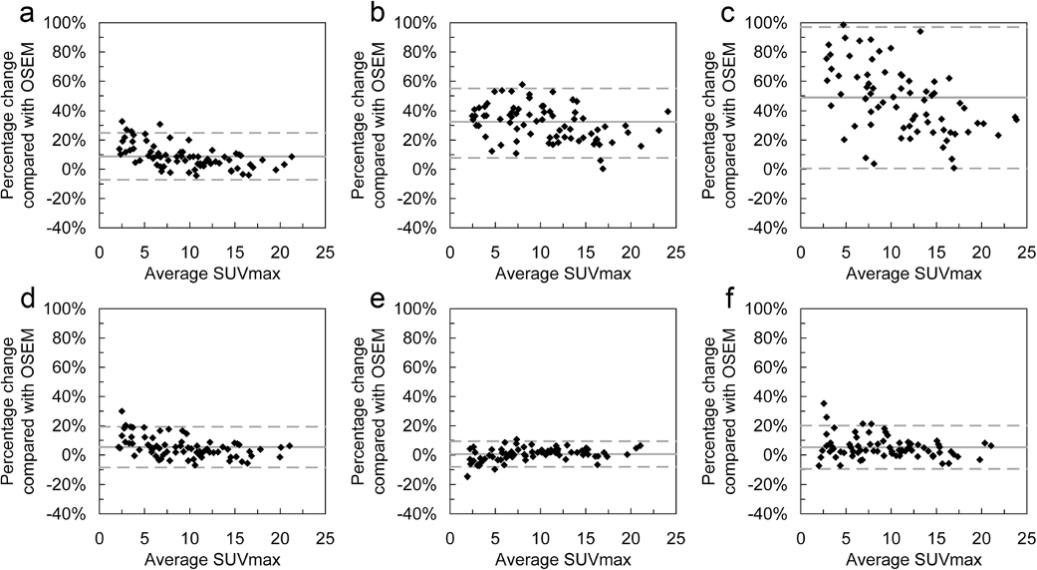
**Bland-Altman plots showing relative percentage differences for SUV**_**max**_ **relative to OSEM.** Plots on the top row are for images with matched image voxel COV post-filters, and plots on the bottom row are for images with matched SUV_max_ recovery post-filters. Reconstructions are TOF **(a, d)**, HD **(b, e)** and UHD **(c, f)**. In each plot, the solid grey line shows the mean percentage difference and the two dashed lines show the 95% confidence intervals.

**Figure 4:**
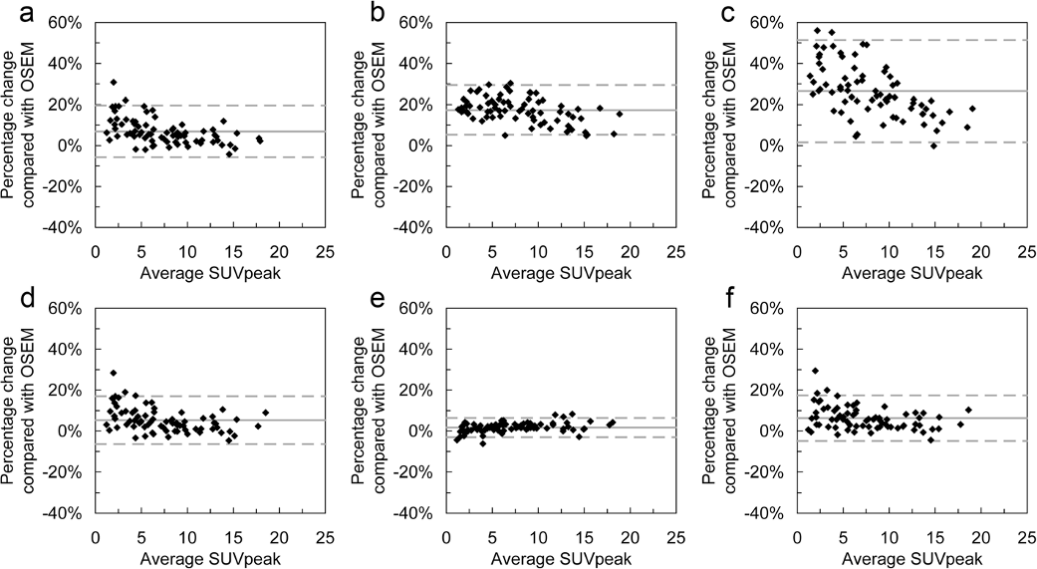
**Bland-Altman plots showing relative percentage differences for SUV**_**peak**_ **relative to OSEM.** Plots on the top row are for images with matched image voxel COV post-filters, and plots on the bottom row are for images with matched SUV_max_ recovery post-filters. Plot layout **(a-f)** and line styles are as per Figure 3.

**Figure 5:**
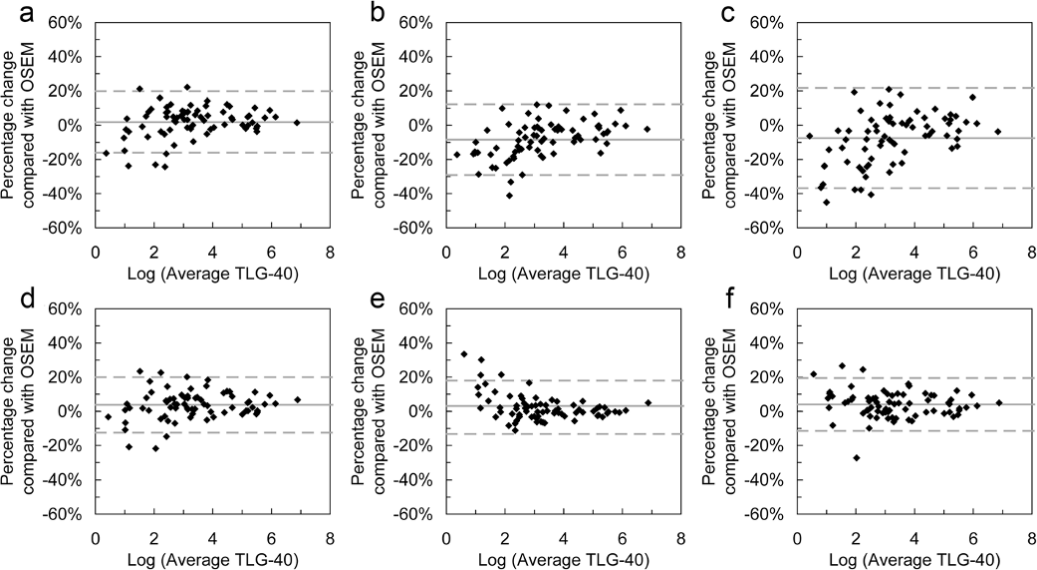
**Bland-Altman plots showing relative percentage differences for TLG-40 relative to OSEM.** Plots on the top row are for images with matched image voxel COV post-filters, and plots on the bottom row are for images with matched SUV_max_ recovery post-filters. Plot layout **(a-f)** and line styles are as per Figure 3.

Tables 4 and 5 summarise the changes of the three uptake measures observed using the PSF and TOF-based reconstructions relative to OSEM. The data in Table 5 for the number of lesions with a change in SUV_max_ greater than 25% occurred in lesions with very low grade uptake (SUV_max_ <2.5). Bland-Altman plots for the relative differences are shown in Figures 3, 4 and 5, which, in addition to data in Tables 4 and 5, show that the smaller values of SUV_max_ and SUV_peak_ experience the greatest increase with matched voxel COV (Figure 3a,b,c and Figure 4a,b,c). For matched SUV_max_ filters, this is still present with TOF algorithms (Figure 3d,f and Figure 4d,f) but not with HD reconstruction.

**Table 4:** **Relative uptake differences for matched voxel COV**

Uptake		TOF	HD	UHD
SUV_max_	Mean% change	+8.8%	+32%	+49%
	95% CI	−7.2% to +25%	+7.8% to +55%	+1.0% to +97%
	*n* > 25% change	4/74	50/74	63/74
SUV_peak_	Mean% change	+6.9%	+17%	+27%
	95% CI	−5.7% to +20%	+5.3% to +29%	+1.6% to +51%
	*n* > 30% change	1/74	1/74	25/74
TLG-40	Mean% change	+1.9%	−8.4%	−7.5%
	95% CI	−16% to +20%	−29% to +12%	−37% to +22%

Mean percentage changes and 95% confidence intervals of the three uptake measures relative to OSEM reconstruction. Also shown are the number of lesions with a greater than 25% and 30% increase in SUV_max_ and SUV_peak_, respectively. Data in the table are from images using post-filters to match image voxel COV.

**Table 5:** **Relative uptake differences for matched SUV**_**max**_ **recovery**

Uptake		TOF	HD	UHD
SUV_max_	Mean% change	+5.4%	+0.6%	+5.3%
	95% CI	−8.5% to +19%	−8.1% to +9.4%	−9.5% to +20%
	*n* > 25% change	1/74	0/74	2/74
SUV_peak_	Mean% change	+5.3%	+1.7%	+6.3%
	95% CI	−6.4% to +17%	−3.0% to +6.5%	−4.9% to +17%
	*n* > 30% change	0/74	0/74	1/74
TLG-40	Mean% change	+3.8%	+2.4%	+4.0%
	95% CI	−13% to +20%	−13% to +18%	−12% to +20%

Mean percentage changes and 95% confidence intervals of the three uptake measures relative to OSEM reconstruction. Also shown are the number of lesions with a greater than 25% and 30% increase in SUV_max_ and SUV_peak_, respectively. Data in the table are from images using post-filters to match SUV_max_.

**Figure 6:**
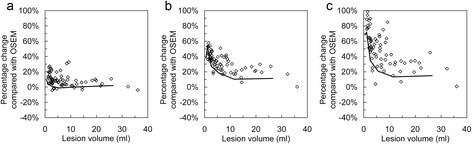
**Percentage differences versus lesion volume.** Relative percentage differences of SUV_max_ for TOF **(a)**, HD **(b)** and UHD **(c)** compared with OSEM for the 74 lung lesions against lesion volume as measured in the OSEM images using a 40% threshold. All plots are for matched voxel COV. The black line illustrates the percentage differences measured for the six spheres of the image quality phantom.

For matched voxel COV, the increase in both SUV_max_ and SUV_peak_ ratio for PSF and TOF-based reconstructions versus OSEM was inversely related to lesion volume as shown in Figure 6. This reflects what was seen in the image quality phantom measurements. The gains in SUV_max_ were most pronounced with UHD, which is likely to be a consequence of reduced post-filtering compared with HD when voxel COV was matched (2.9 mm for UHD and 3.8 mm for HD). Differences in TLG-40 were not dependent on lesion volume. No relationship between SUV difference and lesion volume was observed for matched SUV_max_ post-filters.

Out of the 74 lesions, 59 had a SUV_max_ of >5.0 using OSEM reconstruction. No change to patient management would occur in these instances as a result of an increase of SUV_max_ when using the PSF and TOF-based reconstructions. A key group of ten patients was identified with low or borderline SUV_max_ (<5.0) for suspicion of malignancy using this institute's practice. The SUV_max_ for these 15 lesions in each of the reconstruction algorithms are shown in Table 6. The table shows that, with matched voxel COV, several of these lesions would change classification with HD and UHD, as would be expected from data in previous tables and figures. With matched SUV_max_ filters, there is only one lesion that would have changed classification according to local practice and only with the TOF reconstruction.

**Table 6:** **Changes in SUV**_**max**_ **with lesions with borderline values for malignancy**

Volume	SUV _max_	Matched noise			Matched SUV _max_		
(ml)	OSEM	TOF	HD	UHD	TOF	HD	UHD
1.26	2.1	2.4	*2.8*	*3.6*	2.2	1.8	1.9
9.26	2.2	*2.9*	*2.9*	*4.0*	*2.8*	2.3	*2.9*
2.80	2.2	2.5	*3.2*	*3.6*	2.3	2.2	2.2
1.99	2.4	2.8	*3.2*	*4.2*	2.7	2.2	2.4
6.04	2.5	3.1	*3.3*	*4.3*	3.0	2.7	*3.2*
1.83	2.7	3.4	*3.8*	***5.5***	3.2	2.5	3.0
3.99	2.8	3.1	*3.6*	*4.0*	3.0	2.8	3.0
1.52	3.2	4.0	*4.5*	***6.3***	3.8	2.9	3.4
8.90	3.2	3.6	*4.3*	***5.2***	3.4	3.0	3.3
1.56	3.4	4.0	*4.9*	***6.8***	3.8	3.1	3.6
2.51	3.4	4.2	*4.6*	***6.4***	4.0	3.3	4.0
6.33	3.5	4.0	4.3	**5.3**	3.8	3.4	3.7
1.40	3.9	4.1	***6.0***	***6.9***	3.9	3.8	3.8
14.22	4.4	4.6	4.9	**5.2**	4.5	4.3	4.5
2.06	4.5	**5.6**	***7.0***	***8.5***	**5.4**	4.7	4.2

SUV_max_ values for patients with low uptake (<5.0) for suspicion of malignancy and the SUV_max_ data obtained from the PSF and TOF-based algorithms using the two post-filter strategies. The left column shows the lesion volume as measured from the OSEM PET image using a 40% threshold. Values in bold represent lesions that would have changed classification using a strict SUV_max_ cut-off of 5.0. Values in italics represent increases of greater than 25%.

#### Signal-to-noise gains

Fifty-nine lesions were found to have SUV_max_ above the threshold based on the liver uptake as measured on the OSEM images. Significant SNR_(T-L)_ gains were found for PSF and TOF-based reconstructions with both matched voxel COV and matched SUV_max_. With the addition of PSF modelling, either to OSEM or OSEM + TOF images, there is a more marked gain in SNR_(T-L)_. For matched voxel COV, SNR_(T-L)_ ratios relative to OSEM were 1.10 ± 0.11, 1.43 ± 0.23 and 1.67 ± 0.41 for TOF, HD and UHD, respectively, and for matched SUV_max_, they were 1.19 ± 0.12, 1.58 ± 0.16, and 1.94 ± 0.29, respectively. For each reconstruction algorithm, the improvement in SNR_(T-L)_ with matched SUV_max_ versus matched noise was also significant.

## Discussion

The deployment of PSF and TOF-based reconstruction methods into routine clinical practice for FDG imaging presents a challenge, particularly in centres or collaborative imaging networks with a defined protocol for classification of malignancy based upon SUV data. To our knowledge, this is the first study that has evaluated the performance of PSF and TOF-based reconstruction algorithms with two post-filtering strategies based on the objective criteria of matched image noise (voxel COV) or matched SUV_max_, quantifying the impact on SUV_max_, SUV_peak_, TLG and SNR_(T-L)_. Specific findings are applicable to Siemens HD and ultraHD reconstruction algorithms using the parameters applied in the study.

It is clear from the data in Tables 1 and 2 and Figure 3 that quantification differences occur in the phantom data for all algorithms applied in this study. There are several factors that will contribute to the differences: the effect of statistical noise, partial volume effect, the size (and hence number of voxels) of the region of interest and, for the HD and UHD algorithms, Gibbs artefacts. The contributions from these factors to the measurements of SUV_max_ will differ as reconstruction parameters are varied. We believe that the interactions between the various factors are complex and not completely separable. As such, we do not feel that it is possible to identify one single phenomenon as the source of quantification differences for any of the algorithms used.

It can be seen that overestimation occurs for all four reconstruction algorithms (Table 1) and requires the application of a post-filter to reduce this (Table 2). The smaller filter kernel applied to HD and UHD to match noise combined with voxel correlation leads to a lesser reduction of this overestimation. It can be seen that there appears to be a particular size of object where an overestimation with HD and UHD is particularly prominent with no or minimal levels of post-filtering, which, in part, may be due to overlapping Gibbs edge artefacts. Despite this, it can be seen from HD recovery data in Table 2 that, with matched voxel variance, there is very little dependence of recovery on sphere size for the 13- to 37-mm spheres, which is a desirable property. This highlights the importance of establishing a full understanding of the impact of these algorithms, and it is the duty of medical physics experts to educate clinicians on changes expected to quantification.

Ideally, the implementation of PSF modelling would not lead to Gibbs artefacts, but given the necessary compromises for PET imaging with limited statistics, an improvement in one area such as in image resolution is almost certainly going to lead to a deterioration in other aspects. Overall, whether the changes are desirable is application dependent, with our data showing smaller absolute errors for smaller spheres (but not for large spheres) and reduced dependency on quantification with lesion size.

Matching image noise produces marked increases in SUV_max_, particularly with PSF reconstructions, that are potentially clinically significant, depending on local practice. This highlights the pitfalls of using uptake metrics such as SUV_max,_ that are so sensitive to partial volume effects and reconstruction parameters, with fixed thresholds for malignancy. The largest increases in SUV_max_ occur for small lesions, which typically have low SUV_max_ (less than 5), which is consistent with other studies [36],[37]. One potential solution may be to modify thresholds based on estimated tumour volume. It would be useful to extend the matching of SUV_max_ to smaller objects, but this is not possible due to the limitation of the current NEMA phantom, with 10 mm being the diameter of the smallest sphere insert. It is these small lesions, with SUV_max_ close to the typical cut-offs for discrimination of benign and malignant disease, that are arguably the most critical lesions for lung cancer staging as they are likely to be possible additional pulmonary nodules or lymph nodes. Determining whether a lymph node is malignant, particularly those in the mediastinum, has a considerable influence on the overall staging and will play a major role in patient management. This change in SUV_max_ is expected to require an adaptation of locally used thresholds for discrimination of disease. It was also noted from the phantom studies that variability of SUV_max_ was worse for PSF-based algorithms in the small spheres, which suggests worse test-retest performance in clinical data. This is suspected to be due to increased inter-voxel correlation that is introduced when using PSF-based algorithms [21]. This increased correlation results in a reduction of voxel variance (and hence the voxel COV as used in this study as a noise metric), but it has been shown to potentially result in larger variability of uptake metrics within small ROIs [45]. We feel that the impact of PSF modelling on variability for clinical data has yet to be explored fully, and while this is beyond the scope of this study, it is recommended that caution is observed when applying PSF modelling for assessing response to treatment with follow-up scans. Despite this, the reduced levels of post-filtering required with PSF and PSF + TOF have been shown to improve lesion visualisation [28]-[30].

With matched voxel COV, SUV_peak_ experiences similar differences to those seen for SUV_max_, albeit to a lesser extent. Quantification of peak uptake implicitly includes an additional filtering operation with a spherical kernel. The small mean relative differences for TLG suggests that it is a relatively robust uptake metric when comparing against OSEM images for either filtering strategies. The large degree of variability seen in the relative changes, as highlighted by the confidence intervals in Tables 4 and 5, may be concerning. However, it should also be noted that the total range of TLG observed in this study is approximately a full order of magnitude greater than SUV_max_ and SUV_peak_. The use of TLG has been reported in assessment of therapy response and, recently, for prognosis in a small number of studies. The increased stability of TLG with a volume delineation based on a percentage of SUV_max_ suggests the metric may be more appropriate than SUV_max_ for staging and prognosis as the evidence base for this metric is established. We believe that this is the first time that the dependence of TLG on reconstruction algorithm has been explored in the literature.

Alternatively, post-filters for PSF and TOF-based algorithms can be determined to give SUV_max_ that, according to this institute's practice, would not alter the outcome of the study. For all lesions with borderline SUV_max_ for suspicion of malignancy, relative changes with PSF and TOF-based reconstructions were less than 20%.

Matching SUV_max_ between PSF-based algorithms and OSEM has been demonstrated previously [39]. However, our study has also shown that matching SUV_max_ will significantly reduce the voxel variance in the image compared with OSEM, which we believe has yet to be demonstrated quantitatively. Combined with increased voxel correlation, this reduction of voxel variance alters the image appearance quite considerably and may be perceived as over-smoothing of images. Findings from this study are based upon an image matrix of 256 × 256 voxels, whereas other centres may use different parameters such as 200 × 200 or 400 × 400 voxels, which are common choices on the mCT due to the system's intrinsic 400 × 400 matrix. We believe that, when Gaussian post-filtering is applied, the dependence of both image noise and SUV_max_ on matrix choice is diminished. It has also been shown that the thickness of the walls of the fillable spheres of the NEMA phantom has an impact on SUV_max_ quantification [46],[47]. This is only seen to cause appreciable error with low sphere-to-background contrast and small spheres, and hence, we expect that the impact on the test objects used in this study is likely to be minimal.

It is noted that the degree of post-filtering for the HD and UHD algorithms (6.6 and 6.5 mm, respectively) will reduce spatial resolution for these PSF-based algorithms that are intended to provide superior spatial resolution. However, we feel that this approach may be beneficial when deploying a new PET/CT scanner to an existing clinical setting, comparing patient scans for follow-up with other systems or supporting the transition to a ‘new’ imaging facility with a catalogue or library of images with higher resolution.

In this study, the addition of TOF increased the variation in ratio values of image voxel variance for both phantom and patient data with either matched noise or matched SUV_max_. In the patient data only, TOF appeared to introduce a slight positive bias and greater distribution of differences in the SUV_max_ data. This was not seen in the phantom studies and the cause of this is unclear. It could be due to a dependence on patient size, as TOF is associated with SNR gains proportional to the diameter of object [48]. However, in this study and others [20], this did not appear to apply in lung images where the majority of tissue in the image has low density with very low uptake of FDG.

We believe this is the first study to demonstrate SNR gains with PSF and/or TOF using lesion uptake as a measure of signal with two different criteria for choosing post-filtering. A recent study has shown reductions in voxel variance and gains in SNR but measured only in uniform areas of uptake with patient livers [27]. One study has evaluated SNR gains using lesion uptake as the signal [25] but only comparing images reconstructed with PSF and PSF + TOF, with the intention to demonstrate the SNR gains brought on by TOF. It was expected that SNR gains would be seen for PSF and TOF-based algorithms compared with conventional OSEM. However, it was not anticipated that the gains in SNR would be greater when parameters are chosen to match SUV_max_. This may be of particular relevance for low-contrast lesions elsewhere in the body, such as the abdomen, which do not have the inherent high lesion to background contrast of lung lesions. The notion that increased levels of post-filtering may be superior in terms of SNR gains seems slightly at odds with published work on lesion detection that suggest less post-filtering results in optimal lesion detection [28],[29]. This may be due to fact that the definition of SNR in this study is not a direct indicator of lesion detectability.

There are two limitations with this study where future work is planned. Firstly, no histological correlation with FDG uptake measured in the lesions was performed as in other studies [36]. Therefore, it is not possible to determine cut-off values and diagnostic accuracy of the uptake metrics in the two strategies of implementation. This is arguably outside the scope of this study as the purpose was not to determine such data. Secondly, we have only assessed lung lesions, and from other studies [25], it is likely that reconstruction will perform differently in other areas of the body.

The effect of PSF and TOF-based reconstruction on quantification, particularly SUV_max_, has limited their introduction into routine clinical use despite demonstrated improvements in lesion detectability. This study extends existing studies [39] which have shown that the impact on SUV_max_ can be addressed with appropriate post-filters, by demonstrating that the same approach can be used for reconstructions with TOF reconstructions and also with alternative uptake metrics such as SUV_peak_ or TLG. Furthermore, we have demonstrated that this additional filtering to match SUV_max_ actually provides added gains in SNR over parameters to match image voxel COV. However, if the additional smoothing is visually undesirable, an alternative methodology can be used which performs the additional filtering required to match SUV_max_ only for quantification and is not visualised [41].

## Conclusions

This work evaluated the impact of reconstructions that include PSF modelling and/or TOF on lesion classification according to a local protocol by assessing changes in FDG uptake measurements. Two objective strategies for post-filtering were investigated: matching image voxel COV versus matching SUV_max_. For matched voxel COV, considerable increases in SUV_max_ and SUV_peak_ were observed compared with OSEM. Using post-filters to match SUV_max_ reduced the discrepancies of either SUV_max_ or SUV_peak_ across reconstructions, particularly with PSF modelling. This also resulted in a considerable reduction in voxel variance. Some small discrepancies in patient data still remained when TOF was incorporated, which was not seen in phantom data, warranting further investigation. The TLG metric appears to be more robust in either scheme of post-filtering despite a slightly larger variation in the amount of change, which may be less of a problem considering the large range of TLG data observed. This suggests TLG may be a more suitable metric to adopt instead of SUV_max_ as the evidence base develops. Gains in SNR were seen in both implementations with the greatest gains seen for matched SUV_max_ post-filters.

## Authors’ original submitted files for images

Below are the links to the authors’ original submitted files for images. Authors’ original file for figure 1 Authors’ original file for figure 2 Authors’ original file for figure 3 Authors’ original file for figure 4 Authors’ original file for figure 5 Authors’ original file for figure 6

## Abbreviations

COV: coefficient of variation
EORTC: European Organization for Research and Treatment of cancer
FDG: 2-fluoro-2-deoxy-d-glucose
FWHM: full width at half the maximum
HD: Siemens HD·PET reconstruction
OSEM: ordered subset expectation maximisation
PERCIST: PET response criteria in solid tumours
PET: positron emission tomography
PSF: point spread function
ROI: region of interest
SNR: signal-to-noise ratio
SUV: standardised uptake value
TLG: total lesion glycolysis
TOF: time of flight
UHD: Siemens ultraHD·PET reconstruction
VOI: volume of interest
