# Supplementary figures and images for: Impact of point spread function modelling and time of flight on FDG uptake measurements in lung lesions using alternative filtering strategies
Source: EJNMMI Phys. 2014 Nov 30;1:99. doi: 10.1186/s40658-014-0099-3 (PMC4545221; doi:10.1186/s40658-014-0099-3)

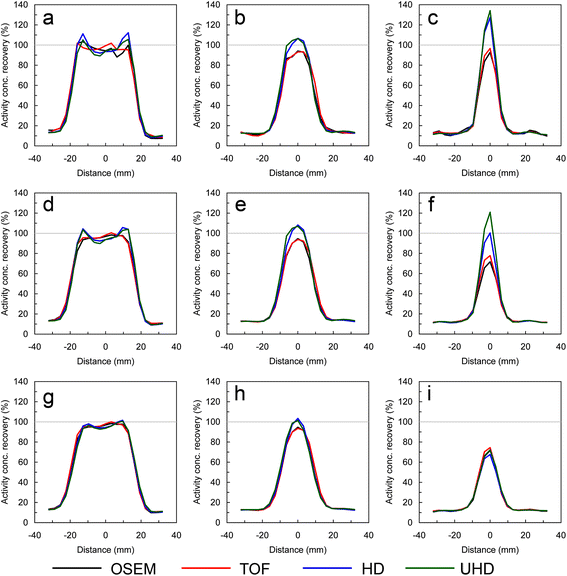

Supplement: Supplementary file 1 — Authors’ original file for figure 1 [file 40658_2014_99_MOESM1_ESM.gif]

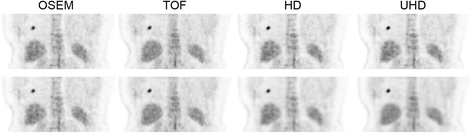

Supplement: Supplementary file 2 — Authors’ original file for figure 2 [file 40658_2014_99_MOESM2_ESM.gif]

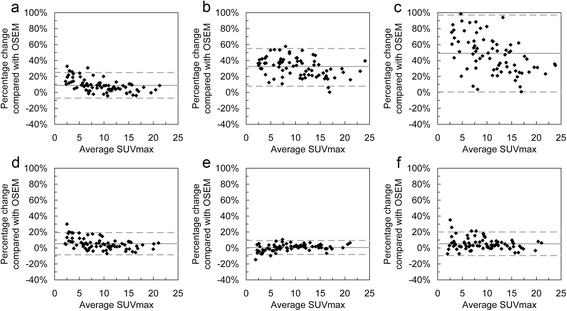

Supplement: Supplementary file 3 — Authors’ original file for figure 3 [file 40658_2014_99_MOESM3_ESM.gif]

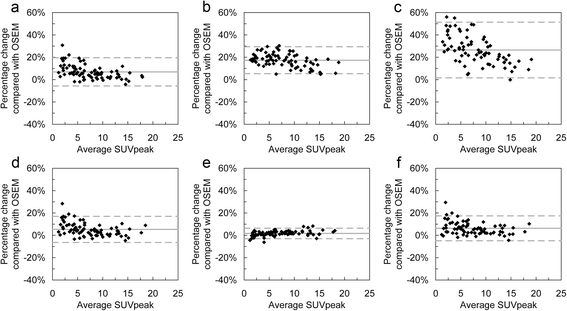

Supplement: Supplementary file 4 — Authors’ original file for figure 4 [file 40658_2014_99_MOESM4_ESM.gif]

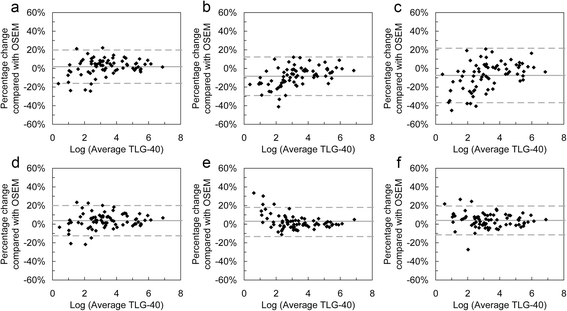

Supplement: Supplementary file 5 — Authors’ original file for figure 5 [file 40658_2014_99_MOESM5_ESM.gif]

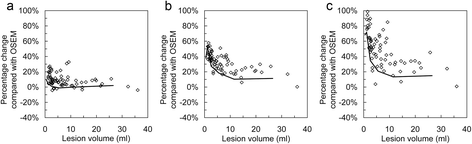

Supplement: Supplementary file 6 — Authors’ original file for figure 6 [file 40658_2014_99_MOESM6_ESM.gif]
